# Case Report: Postmenopausal hyperandrogenism misled by adrenal incidentaloma: a rare case of androgen-secreting ovarian adult granulosa cell tumor and clinical implications

**DOI:** 10.3389/fonc.2025.1732541

**Published:** 2026-01-12

**Authors:** Conghui Cao, Xiaoli Wang

**Affiliations:** Department of Endocrinology and Metabolism, Institute of Endocrinology, National Health Commission (NHC) Key Laboratory of Diagnosis and Treatment of Thyroid Diseases, The First Hospital of China Medical University, Shenyang, China

**Keywords:** adult-type granulosa cell tumor, alopecia, androgen-secreting tumor, case report, postmenopausal hyperandrogenism

## Abstract

**Background:**

Ovarian adult-type granulosa cell tumors (AGCTs) are rare sex cord-stromal neoplasms, accounting for 95% of ovarian granulosa cell tumors (GCTs) but only 10% of which secrete androgens. For postmenopausal women, hyperandrogenism is commonly attributed to conditions like polycystic ovary syndrome (PCOS), while androgen-secreting AGCTs are extremely rare, often leading to diagnostic challenges—especially when accompanied by adrenal incidentalomas that may divert clinical attention.

**Case presentation:**

This study presents a case of a 57-year-old postmenopausal Chinese woman (menopause at 48) with 5 years of progressive hyperandrogenic symptoms (facial/perianal/leg hirsutism, vertex/frontal alopecia, deepened voice, clitoral hypertrophy). Initial evaluation revealed a left adrenal nodule and elevated testosterone; however, pelvic ultrasound (limited by bowel gas) showed no obvious abnormalities in the ovaries. Further tests at the First Affiliated Hospital of China Medical University confirmed markedly elevated serum testosterone (37.00 nmol/L, ref: 0.69-1.49 nmol/L) and free testosterone (300.90 pmol/L, ref: 0.77-33.03 pmol/L), suppressed LH/FSH, low AMH (0.01 ng/mL), and a 2.6×2.0 cm enhancing nodule in the right adnexa on pelvic MRI. Notably, adrenal-derived androgens were within normal ranges in this case, ruling out adrenal origin of hyperandrogenism. She underwent laparoscopic total hysterectomy + bilateral salpingo-oophorectomy; permanent pathological examination (with immunohistochemistry: Vimentin+, α-inhibin+, Calretinin+, Ki67+ ~2%) confirmed FIGO Stage IA AGCT (frozen section initially suggested benign tissue). Postoperatively, her androgen levels normalized, hirsutism/oily skin/acne improved, blood pressure decreased, though voice deepening persisted.

**Discussion:**

This case underscores critical clinical lessons: Postmenopausal women with severe hyperandrogenism require comprehensive adrenal and pelvic evaluation (prioritizing MRI over ultrasound due to higher sensitivity for small tumors) even with adrenal findings. Comprehensive hormonal profiling (testosterone, adrenal androgens, gonadotropins, AMH) aids in distinguishing tumorous from non-tumorous causes, and permanent pathology is essential to avoid misdiagnosis from frozen sections. Surgical resection (bilateral salpingo-oophorectomy for postmenopausal patients) is effective for AGCT management, and long-term follow-up is crucial given the risk of late recurrence. Additionally, AGCT diagnosis is feasible in institutions without inhibin B/genetic testing via integrated clinical, hormonal, and imaging data. This case aims to raise awareness of rare androgen-secreting AGCTs to reduce diagnostic delays and improve management.

## Introduction​

Ovarian granulosa cell tumors (GCTs) are rare sex cord-stromal neoplasms, accounting for 2-3% of all ovarian cancers, with adult-type GCTs (AGCTs) comprising 95% of GCTs (1–3). Most AGCTs secrete estrogen, leading to postmenopausal bleeding or endometrial hyperplasia, while only 10% produce androgens, manifesting as hirsutism, clitoral hypertrophy, deepened voice, or alopecia (3). For postmenopausal women, the most common cause of absolute androgen excess is polycystic ovary syndrome (PCOS), which typically induces mild-to-moderate hyperandrogenic symptoms. Androgen-secreting ovarian tumors are extremely rare, occurring in 1–3 per 1000 patients presenting with hirsutism and accounting for <0.5% of all ovarian tumors (4). Androgen-secreting AGCTs are even more uncommon.

Diagnosis is often complicated by overlapping hormonal and imaging features between AGCTs and non-tumorous conditions such as ovarian hyperthecosis (OHT) (5). Additionally, adrenal incidentalomas—with a prevalence of up to 7% in elderly populations (6) —may divert attention from ovarian evaluation. This case of a postmenopausal woman with hyperandrogenism and adrenal findings highlights the importance of comprehensive workup, including pelvic imaging and hormonal correlation, to avoid misdiagnosis of rare androgen-secreting AGCTs.

## Case presentation​

**Figure 1:**
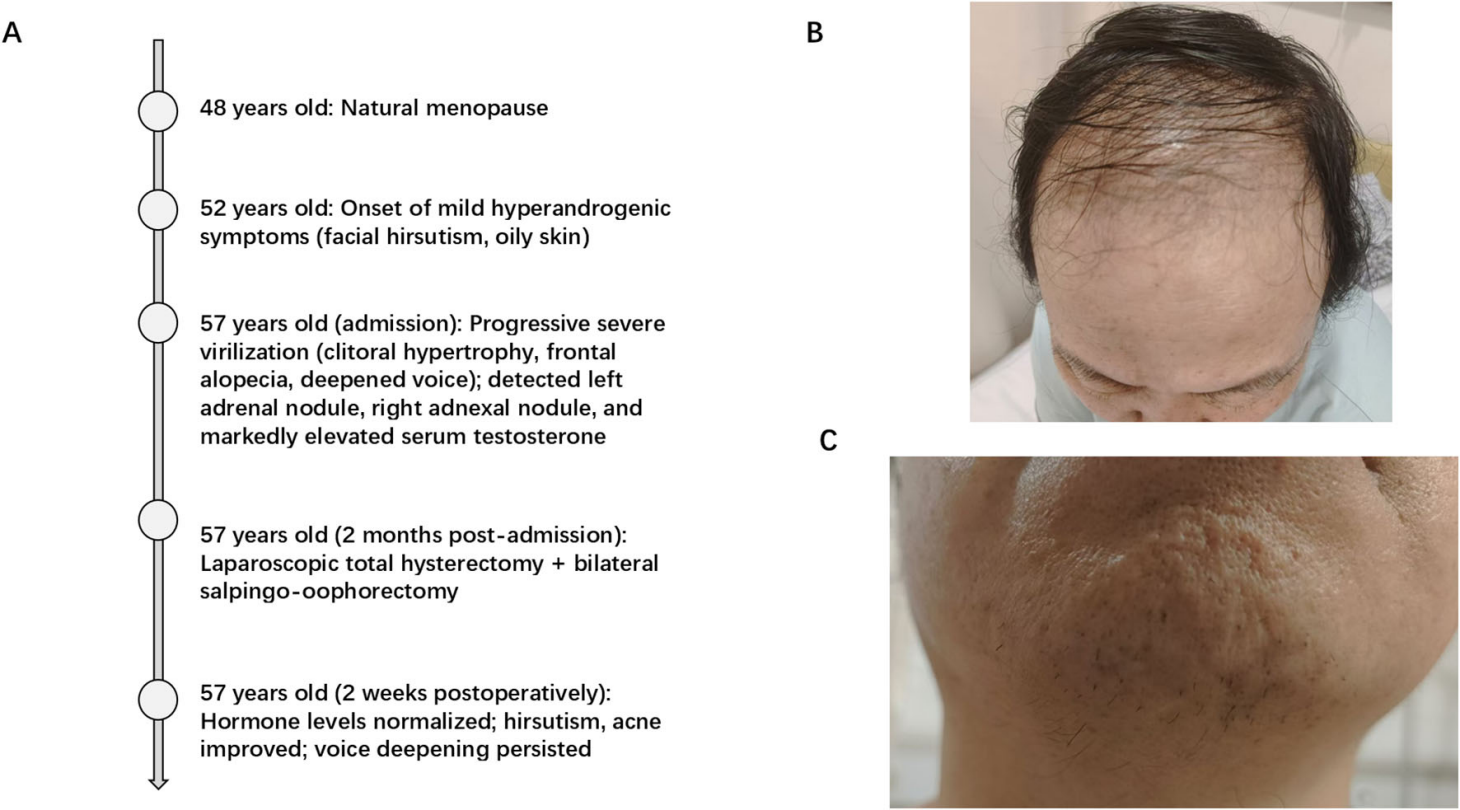
Clinical timeline and manifestations of hyperandrogenism in the patient. **(A)** Clinical timeline. **(B)** Vertex alopecia (Ludwig grade III) was observed, characterized by significant hair thinning at the top and front of the scalp. **(C)** Oily facial skin and visible mandibular hirsutism (patient with regular shaving history).

A 57-year-old Chinese woman was admitted to the First Affiliated Hospital of China Medical University. She had been menopausal since 48 and reported 5-year progressive symptoms (**Figure 1**): facial, perianal, and leg hirsutism; vertex and frontal scalp alopecia; deepened voice; and clitoral hypertrophy. She denied abdominal pain, vaginal bleeding, or discharge.

Physical examination showed blood pressure 150/90 mmHg, heart rate 76 beats/min, temperature 36.5°C, and body mass index (BMI) 26.91 kg/m². Dermatological findings included marked facial hirsutism, vertex and frontal alopecia [Ludwig grade III (7)], oily facial skin, acne, and clitoral hypertrophy (~2 cm) (**Figure 1**). Abdomen was soft, non-tender, with no palpable masses; bimanual exam revealed non-palpable uterus (postmenopausal) and no adnexal masses. Other systems showed no abnormalities.

### Laboratory tests

Thyroid function showed mildly elevated TSH (6.26 mIU/L; ref: 0.35-4.94), normal free triiodothyronine (FT3: 3.4 pmol/L; ref: 2.63-5.7 pmol/L), normal free thyroxine (FT4: 12.3 pmol/L; ref: 9.01-19.05 pmol/L), and elevated antibodies (TPOAb 448.98 IU/mL, TGAb 179.92 IU/mL), consistent with autoimmune thyroiditis and subclinical hypothyroidism. Hormonal markers (preoperative and postoperative) are summarized in **Table 1**: preoperative serum testosterone and free testosterone were markedly elevated; anti-Müllerian hormone (AMH) was markedly low (0.01 ng/mL); estradiol (E2) was slightly elevated; and luteinizing hormone (LH) and follicle-stimulating hormone (FSH) were profoundly suppressed. A serum testosterone level >5 nmol/L is a key indicator suggesting androgen-secreting neoplasms in postmenopausal women.

**Table 1:** Hormonal testing before and after surgery​.

Hormone	Preoperative value	Postoperative value	Reference Range
T (nmol/L)	37.00	<0.69	0.69-1.49
FT (pmol/L)	300.90	5.18	0.77-33.03
E2 (pmol/L)	221.00	119.00	73.4-110
17α-OHP (ng/mL)	1.06	0.52	0.07-0.89
A4 (nmol/L)	11.10	3.20	1.05-11.52
DHEAS (μmol/L)	4.89	3.15	0.96-11.67
LH (mIU/mL)	0.37	7.40	11.3-39.8
FSH (mIU/mL)	0.80	15.80	21.7-153
SHBG (nmol/L)	19.30	25.70	18-144
AMH (ng/mL)	0.01	0.01	0.00-1.15
TSH (mIU/L)	6.26	5.83	0.35-4.94
fT4 (pmol/L)	11.87	10.72	9.01-19.05
fT3 (pmol/L)	4.36	3.72	2.63-5.7
8 AM ACTH (pg/mL)	15.87	–	7.2-63.3
15 PM ACTH (pg/mL)	15.21	–	7.2-63.3
24 PM ACTH (pg/mL)	3.25	–	7.2-63.3
8 AM COR (nmol/L)	209.10	–	133.00-537.00
15 AM COR (nmol/L)	116.10	–	68.2-327.00
24 AM COR (nmol/L)	28.73	–	68.2-327.00

T, testosterone; FT, free testosterone; E2, estradiol; 17α-OHP, 17α-Hydroxyprogesterone; A4, androstenedione; DHEAS, dehydroepiandrosterone sulfate; LH, luteinizing hormone; FSH, follicle-stimulating hormone; SHBG, sex hormone-binding globulin; AMH, anti-Müllerian hormone; TSH, thyrotropin-releasing hormone; fT4, free thyroxine; fT3, free triiodothyronine; ACTH, adrenocorticotropic hormone; COR, cortisol.

ACTH-cortisol rhythm was measured, with results showing normal rhythm and levels: 8 AM cortisol 280 nmol/L, 4 PM 160 nmol/L, and 12 AM 28.73 nmol/L. Adrenal-derived androgens, including DHEAS and androstenedione (A4), were within normal ranges. Cancer antigen 125 (CA125), carcinoembryonic antigen (CEA), alpha-fetoprotein (AFP), CA153, and CA199 were within normal ranges. Biochemistry: Triglycerides (2.9 mmol/L; reference range: 0.00–1.70 mmol/L) were elevated, and high-density lipoprotein (HDL: 0.82 mmol/L; reference range: 0.91–1.92 mmol/L) was decreased; liver/kidney function and uric acid were normal.

### Imaging

**Figure 2:**
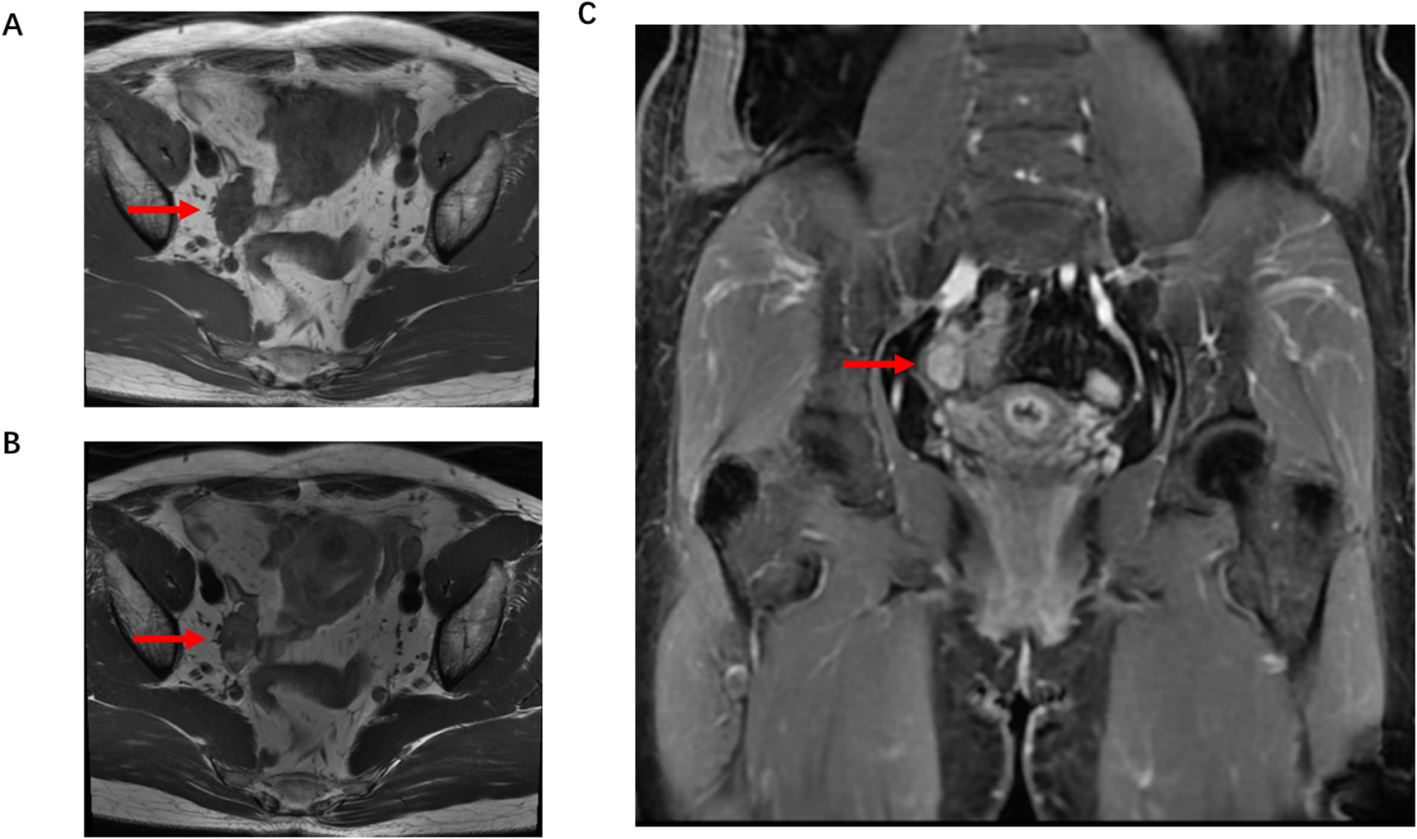
Pelvic contrast-enhanced magnetic resonance imaging (MRI) of the patient. **(A)** Axial T1-weighted image showed a nodular lesion in the right adnexa with a long T1 signal (arrow). **(B)** Axial T2-weighted image revealed a mixed T2 signal in the right adnexal nodule (arrow), measuring approximately 2.6×2.0 cm in size. **(C)** Coronal contrast-enhanced T1-weighted image demonstrated obvious enhancement of the right adnexal nodule (arrow), indicating increased vascularity. No abnormal signals or enhancing lesions were detected in the left adnexa. This imaging feature was highly suggestive of an ovarian neoplasm, which was later confirmed as AGCT.

Adrenal enhanced CT showed a left adrenal junction oval low-density nodule (1.0×0.7 cm, CT value 27 HU; arterial phase 110 HU, delayed phase 58 HU), consistent with hyperplasia or small adenoma. Pelvic ultrasound (limited by bowel gas) showed an atrophic uterus, right ovary 2.90×1.52 cm, left ovary 1.94×1.16 cm, and no obvious masses. Pelvic enhanced MRI detected a right adnexal 2.6×2 cm enhancing nodule (long T1, mixed T2 signal) and a uterine anterior wall 3.1×2.5 cm submucosal leiomyoma (T1 isointense, T2 hypointense) (**Figure 2**).

### Diagnostic workflow and result interpretation

The patient’s severe hyperandrogenic symptoms (clitoral hypertrophy, frontal alopecia, deepened voice) combined with markedly elevated testosterone (37.00 nmol/L, 25× upper reference limit) and free testosterone (300.90 pmol/L) strongly suggested an androgen-secreting neoplasm. Suppressed LH/FSH indicated with severe androgen excess with non-PCOS pathology. Normal adrenal androgens (DHEAS, A4) and ACTH-cortisol rhythm ruled out adrenal origin of hyperandrogenism. Pelvic MRI identified a right adnexal nodule (2.6×2.0 cm) consistent with an ovarian tumor, while ultrasound limitations (bowel gas) were noted. Collectively, these findings led to the preliminary diagnosis of an ovarian androgen-secreting tumor.

Given the high suspicion for an ovarian sex cord-stromal tumor (AGCT), surgical resection was recommended to: (1) confirm the pathological diagnosis; (2) eliminate autonomous androgen secretion; and (3) reduce recurrence risk—aligning with management guidelines for suspected ovarian hormone-secreting tumors (8).

**Figure 3:**
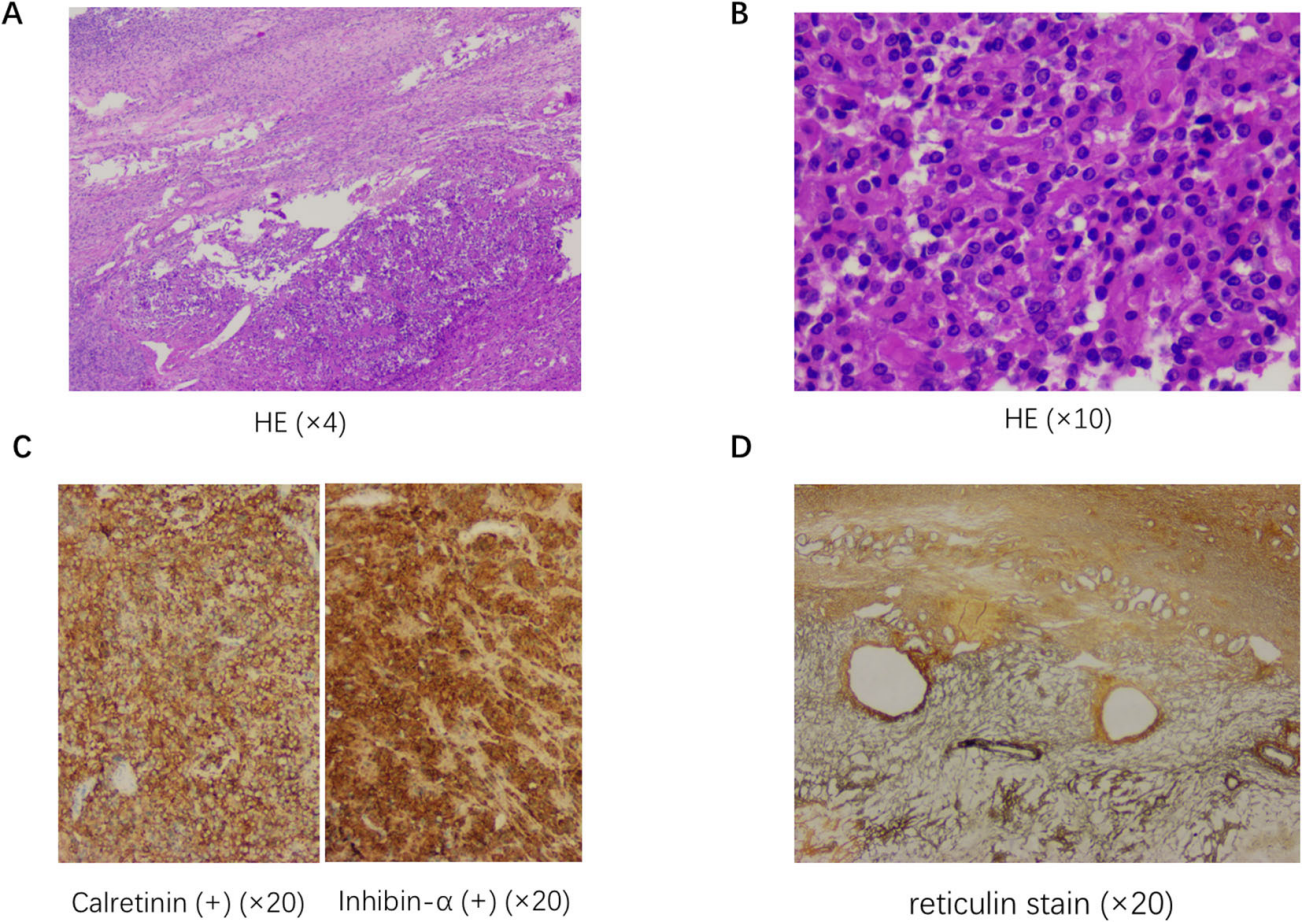
Histopathological and immunohistochemical findings of the ovarian adult-type granulosa cell tumor (AGCT). **(A, B)** Hematoxylin and eosin (HE) staining at low magnification [×4, **(A)**] and medium magnification [×10, **(B)**] showed the tumor tissue with an insular trabecular growth pattern. The tumor cells exhibited the characteristic “coffee bean” morphology, which is pathognomonic for AGCT—manifested as longitudinal nuclear grooves. **(C)** Immunohistochemical staining (×20) showed positive expression of Calretinin (left panel) and Inhibin-α (right panel) in the tumor cells, which are specific markers for sex cord-stromal tumors and further confirmed the diagnosis of AGCT. **(D)** Reticulin staining (×20) revealed a delicate reticular fiber network surrounding the tumor cell nests, a typical histological feature of AGCT.

Two months later, she underwent laparoscopic total hysterectomy + bilateral salpingo-oophorectomy at a local hospital. Intraoperative findings: A 2.0×2.0 cm cystic mass was identified in the right ovary; the left ovary and uterus were normal; no ascites or peritoneal seeding was observed. Frozen section suggested benign ovarian tissue, but permanent pathological examination confirmed AGCT (gross: 2.0×2.0 cm cyst with solid components; microscopy: insular trabecular pattern, “coffee-bean” nuclear grooves; immunohistochemistry: Vimentin+, α-inhibin+, Calretinin+, CD56+, CD99+, Ki67+ ~2%) (**Figure 3**).

Diagnoses: Ovarian adult-type granulosa cell tumor (AGCT, FIGO Stage IA), AGCT-induced postmenopausal hyperandrogenism, autoimmune thyroiditis.

Postoperatively, the patient recovered uneventfully and was discharged on postoperative day 5. At the 2-week follow-up: All hormonal markers normalized (**Table 1**); hirsutism improved (no longer requiring facial shaving); oily skin and acne resolved; alopecia slightly improved; voice deepening persisted; and blood pressure decreased to 120/70 mmHg. The follow-up plan includes monthly assessments for the first 3 months, quarterly evaluations for the next 2 years, semi-annual surveillance for the subsequent 3 years, and annual follow-up thereafter (total duration ≥15 years), including routine gynecological examinations, pelvic ultrasound (supplemented with chest X-ray/abdominal CT as needed), and serum marker detection (androgen profile, estradiol, AMH) (9).

## Discussion​

Androgen-secreting AGCTs account for approximately 10% of all GCTs, presenting unique clinical challenges. Unlike estrogen-secreting AGCTs—which often cause postmenopausal bleeding, endometrial hyperplasia, or endometrial cancer—androgen-secreting AGCTs induce virilization, with symptoms progressing gradually and leading to diagnostic delays (2, 3). Our patient’s 5-year symptom-to-diagnosis interval reflects low clinical suspicion for ovarian tumors in postmenopausal hyperandrogenism (2). Notably, the patient had both vertex and frontal alopecia, a common but underrecognized feature of severe hyperandrogenism that should prompt clinicians to consider androgen-secreting neoplasms.

Notably, hyperandrogenic manifestations of androgen-secreting tumors may relate to peripheral aromatization of testosterone. This patient had slightly elevated preoperative estradiol and a 3.1×2.5 cm submucosal uterine leiomyoma on MRI—findings supporting this aromatization process. Excess tumor-derived testosterone can convert to estrogen peripherally, and sustained estrogen exposure may promote leiomyoma growth in postmenopausal women (10).

Adrenal enhanced CT and pelvic ultrasound were performed almost simultaneously during the patient’s initial evaluation, which revealed a left adrenal nodule—highlighting a common clinical pitfall. Adrenal incidentalomas are frequent in older adults but rarely explain severe hyperandrogenism unless associated with elevated DHEAS (11). Here, preoperative adrenal androgens (androstenedione 11.10 nmol/L, DHEAS 4.89 μmol/L) were within reference ranges, and postoperative levels remained normal—ruling out adrenal origin (6). Comprehensive biochemical evaluations were conducted to exclude other adrenal-related differential diagnoses of hyperandrogenism: Cushing’s syndrome was ruled out by a normal ACTH-cortisol rhythm (especially nocturnal cortisol 28.73 nmol/L), absence of typical clinical manifestations (12); congenital adrenal hyperplasia (e.g., CYP11 deficiency) was excluded by the presence of elderly onset, normal adrenal androgens, normal 17-hydroxyprogesterone, and lack of electrolyte abnormalities; primary aldosteronism is of low likelihood due to the absence of hypokalemia and postoperative blood pressure normalization without antihypertensive medication, though long-term monitoring of blood pressure, serum potassium, and aldosterone/renin ratio (ARR) is recommended.

Diagnosing androgen-secreting AGCTs requires integrating clinical, hormonal, and imaging data. Most previously reported androgen-secreting AGCT cases in postmenopausal women presented with rapid virilization (1–2 years) and elevated AMH (13). In contrast, our patient’s 5-year indolent symptom course and low AMH (0.01 ng/mL) are rarely documented—this expands the clinical spectrum of androgen-secreting AGCTs. Notably, the differentiation between androgen-secreting AGCTs and OHTs can be particularly challenging when tumor biological behavior is indolent. As established in clinical practice guidelines (12), in perimenopausal or postmenopausal women, a slow progression of virilizing symptoms suggests the possibility of OHT; while an early onset of mild to moderate hyperandrogenic symptoms with slow progression is more consistent with PCOS; and a late onset of virilizing symptoms with rapid progression indicates a potential hormone-secreting tumor. In this case, the patient’s androgenic signs developed progressively over 5 years, without the rapid progression typical of aggressive androgen-secreting tumors. This clinical course highlights that relatively indolent androgen-secreting AGCTs can have overlapping manifestations with OHT, making clinical differentiation difficult. Suppressed LH/FSH indicated with severe androgen excess with non-PCOS pathology. In women with OHT, gonadotropins are typically not suppressed and even in androgen-secreting ovarian tumors levels are often observed in the postmenopausal range (14). At this juncture, androgen levels become a critical discriminator: serum testosterone >5 nmol/L is a key red flag for neoplasm or OHT, with higher levels more suggestive of tumors (12). Our patient’s preoperative 37.00 nmol/L level—25 times the upper reference limit—strongly suggested a tumor.

The 96-h dexamethasone suppression test is a valuable tool for distinguishing adrenal vs. ovarian sources of hyperandrogenism—adrenal-derived androgens typically show significant suppression after dexamethasone administration, while ovarian-derived androgens remain unaffected due to autonomous secretion (15). Although this test was not performed in our patient, the combination of normal adrenal androgens, pelvic MRI findings of an ovarian nodule, and postoperative normalization of androgens sufficiently confirmed the ovarian origin of hyperandrogenism. This test may be considered in cases with ambiguous hormonal or imaging results to further clarify the source of androgen excess.

Preoperative AMH in this patient was markedly low (0.01 ng/mL), consistent with postmenopausal ovarian reserve depletion. While AMH is typically elevated in AGCTs (13), our patient’s low AMH suggests that not all AGCTs present with increased AMH. Nevertheless, this finding helped rule out conditions such as PCOS (which typically presents with normal or elevated AMH in premenopausal women) and confirmed postmenopausal status, reinforcing the need to investigate unexpected hyperandrogenism in this population.

Inhibin B—a glycoprotein hormone specifically secreted by granulosa cells—has been established as a highly sensitive and specific marker for AGCTs (16); additionally, detection of FOXL2 gene mutations in circulating tumor DNA (ctDNA) or tumor tissue is a key diagnostic marker for GCTs (17, 18). However, inhibin B testing and genetic testing were unavailable at our hospital, so we relied on comprehensive hormonal profiling and pelvic MRI to guide diagnosis—an approach that remains feasible for institutions without access to specialized tumor markers.

Imaging plays a critical role: pelvic ultrasound is often limited by bowel gas (as in this case), while MRI offers 78% sensitivity for detecting small ovarian tumors (2)—making it the preferred modality for unexplained hyperandrogenism. Notably, ovarian asymmetry on ultrasound should raise suspicion for ovarian tumors (19). Pathological confirmation remains the gold standard: AGCTs are distinguished by “coffee-bean” nuclear grooves and positive IHC for α-inhibin/Calretinin (17). Our patient’s Ki67 index (~2%) reflects the indolent nature of AGCTs, consistent with their low malignant potential. However, frozen section inaccuracy (benign vs. malignant AGCT) underscores the need for permanent pathology to avoid undertreatment.

Surgical resection is the cornerstone of AGCT management (12). For postmenopausal patients, bilateral salpingo-oophorectomy is standard, as it eliminates androgen secretion—evidenced by the patient’s normalized testosterone and free testosterone (**Table 1**)—and reduces recurrence risk (17). Hysterectomy was added here due to uterine leiomyoma, though not mandatory for AGCTs. Fertility-sparing surgery (unilateral salpingo-oophorectomy) is an option for premenopausal women with Stage IA disease, but our patient had no fertility needs.

The diagnostic challenge posed by indolent androgen-secreting AGCTs also underscores the need for long-term postoperative follow-up—not to distinguish residual/recurrent tumor from non-tumorous hyperandrogenism (which resolved postoperatively), but to monitor for tumor recurrence. Postoperative outcomes are favorable for Stage IA AGCTs. Our patient’s improved hirsutism and normalized androgens confirm successful tumor resection, though persistent voice deepening is common—attributed to irreversible vocal cord changes from long-term androgen exposure. Postoperative estradiol (119.00 pmol/L) remained slightly above the reference range, likely due to peripheral conversion of adrenal androgens, which is clinically insignificant. Long-term follow-up is critical: AGCTs have a unique pattern of late recurrence, and 80% of recurrent cases are fatal (17). Key recurrence risk factors for AGCTs include incomplete resection, high Ki67 index, and advanced stage—none of which are present in this patient, so her recurrence risk is relatively low. Postoperatively, inhibin B levels (if measurable) would be expected to normalize rapidly (within 2–4 weeks); persistently elevated levels would raise concern for residual tumor or recurrence. Given that inhibin B testing is unavailable at our hospital, we will rely on serial clinical exams, pelvic imaging, and sex hormone monitoring for surveillance.

This case offers key clinical lessons. First, postmenopausal women with new-onset hyperandrogenism—especially severe virilization (clitoral hypertrophy, frontal alopecia, deepened voice)—require both adrenal and pelvic evaluation, regardless of adrenal findings. Second, comprehensive hormonal profiling (including testosterone, adrenal androgens, gonadotropins, and AMH) is critical to distinguish tumorous vs. non-tumorous causes; the stark contrast between our patient’s preoperative and postoperative hormone levels reinforces the value of serial testing, even without inhibin B. Third, MRI should be prioritized over ultrasound for pelvic evaluation in unexplained hyperandrogenism, given its higher sensitivity for small tumors. Additionally, this case highlights the feasibility of AGCT diagnosis in institutions without inhibin B testing, using alternative tools like hormone panels and MRI. Future efforts to expand access to inhibin B testing in resource-limited settings could further improve diagnosis, but our experience demonstrates accurate diagnosis remains achievable with existing modalities.

By raising awareness of androgen-secreting AGCTs, this case aims to reduce diagnostic delays and improve management of rare ovarian tumors in postmenopausal hyperandrogenism. Limitations of this case include the lack of inhibin B testing and FOXL2 mutation analysis, which are considered specific markers for AGCTs. Additionally, the follow-up duration is short—long-term data will be needed to confirm the absence of residual tumor or recurrence. Despite these limitations, the integrated use of clinical symptoms, hormonal profiling, and MRI still enabled accurate diagnosis, providing a feasible approach for institutions without access to specialized testing. Future research should explore targeted therapies for recurrent AGCTs—an area where current evidence remains limited.

## Data Availability

The raw data supporting the conclusions of this article will be made available by the authors, without undue reservation.
